# Targeting the Host Response: Can We Manipulate Extracellular Matrix Metalloproteinase Activity to Improve Influenza Virus Infection Outcomes?

**DOI:** 10.3389/fmolb.2021.703456

**Published:** 2021-07-05

**Authors:** Jess Pedrina, John Stambas

**Affiliations:** Faculty of Health, School of Medicine, Deakin University, Geelong, VIC, Australia

**Keywords:** immunity, extracellular matrix, influenza, therapeutics, metalloproteinases

## Abstract

Each year, hundreds of thousands of individuals succumb to influenza virus infection and its associated complications. Several preventative and therapeutic options may be applied in order to preserve life. These traditional approaches include administration of seasonal influenza vaccines, pharmacological interventions in the form of antiviral drug therapy and supportive clinical approaches including mechanical ventilation and extracorporeal membrane oxygenation. While these measures have shown varying degrees of success, antiviral therapies and vaccination are constrained due to ongoing antigenic drift. Moreover, clinical approaches can also be associated with complications and drawbacks. These factors have led to the exploration and development of more sophisticated and nuanced therapeutic approaches involving host proteins. Advances in immunotherapy in the cancer field or administration of steroids following virus infection have highlighted the therapeutic potential of targeting host immune responses. We have now reached a point where we can consider the contribution of other “non-traditional” host components such as the extracellular matrix in immunity. Herein, we will review current, established therapeutic interventions and consider novel therapeutic approaches involving the extracellular matrix.

## Introduction

**FIGURE 1:**
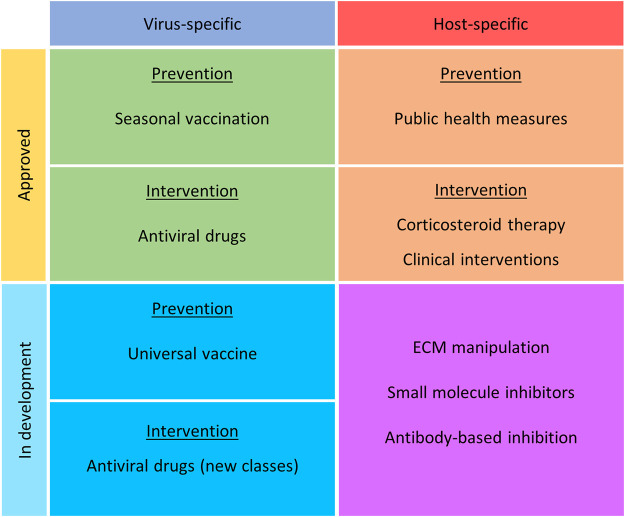
Current and emerging intervention strategies for the prevention and treatment of influenza virus infection. Strategies have been noted as virus or host specific, approved or in development, and nature of treatment (prevention or intervention).

Influenza virus infections, known colloquially as “the flu” are acute infections of the respiratory tract caused by members of the influenza virus genera in the *Orthomyxoviridae* family. Though influenza A, B, and C viruses are capable of infecting humans, seasonal epidemics are typically caused by influenza A and B viruses (Kawaoka and Neumann, 2012). At the moment, only influenza A viruses are considered to have pandemic potential. While the majority of these infections are self-limiting in healthy individuals, when susceptible members of the population become infected, the prognosis may be far less favourable. Those particularly at risk of influenza virus complications are predominantly the very young, the elderly and the immunocompromized. When these individuals become infected, there is a significantly greater likelihood of complications including viral pneumonia, secondary bacterial pneumonia, acute respiratory distress syndrome (ARDS), sepsis, encephalitis, pericarditis, and myocarditis, just to name a few (reviewed by (Kalil and Thomas, 2019)). It is in these instances that medical intervention is required. In addition to the risk posed by seasonal influenza virus epidemics to vulnerable populations, it is also vital that we have effective intervention strategies available in the event of an influenza virus pandemic. As demonstrated by the 1918 “Spanish Flu,” the impact on the the aforementioned susceptible populations was less Hoffman (2011), Short et al. (2018), Auladell (2019) than that seen on younger, healthy members of the population. Furthermore, the ongoing COVID-19 pandemic has highlighted global vulnerabilities when dealing with large-scale respiratory virus outbreaks. It is therefore necessary to re-evaluate current strategies, whilst also developing alternative approaches that can be applied to a variety of infectious agents. This will be discussed throughout this review. A summary of current and emerging strategies can be found in Figure 1 and Table 1.

**TABLE 1:** Evaluation of current and emerging therapeutics for influenza virus infection.

Strategy	Current/emerging	Prophylactic/therapeutic	Strengths	Limitations
Seasonal vaccination	Current	Prophylactic	Offers a range of protection against seasonal strains of influenza virus (Hoft et al. (2011), Estrada and Schultz-Cherry (2019)	Negatively impacted by antigenic drift and shift Demicheli et al. (2018), Sautto et al. (2018), Estrada and Schultz-Cherry (2019)
Universal vaccination	Emerging	Prophylactic	Antigenic drift has less impact and can be applied to pandemic strains Ellebedy and Webby (2009), Ellebedy et al. (2014), Jang and Seong (2019)	Still under development and uncertainty remains regarding effectiveness Estrada and Schultz-Cherry (2019)
Public health measures	Current	Prophylactic	Reduces the transmission of influenza virus Yeoh et al. (2020)	Not socially or economically sustainable Dubey et al. (2020)
Antiviral drug therapy	Current	Both	May be used in a prophylactic or therapeutic context Amarelle et al. (2017), Higashiguchi et al. 2018, Gaitonde et al. (2019)	Emergence of antiviral drug resistance that limits efficacy Lackenby et al. (2011), Hussain et al. (2017), Goldhill et al. (2018)
Corticosteroid therapy	Current	Therapeutic	May reduce lung pathology Li et al. (2017)	May contribute to increased mortality Viasus et al. (2011), Ni et al. (2019), Ye et al. (2020), Zhou et al. (2020)
Clinical interventions	Current	Therapeutic	Potentially lifesaving Davies et al. (2009), Popat and Jones (2012)	Invasive, potential for increased viral transmission Davies et al. (2009), Arulkumaran et al. (2020), Ng et al. (2020), Al Lawati et al. (2021)
ECM manipulation	Emerging	Unknown	Newly identified roles for zinc proteases in virus infection. Wu et al. (2007), McMahon, et al. (2016), Rojas-Quintero et al. (2018), Boyd et al. (2020)	New field of research – many unknowns
Small molecule inhibitors	Emerging and current (in other disorders)	Unknown	Evidence of successful MMP, ADAM and ADAMTS protein inhibition Malemud (2019), Santamaria et al. (2021)	Pre-clinical stages
Antibody-based inhibition	Emerging and current (in other disorders)	Unknown	Evidence of *in vitro, in vivo* and human *ex vivo* inhibition Larkin et al. (2015), Santamaria et al. (2015)	May lack binding specificity Santamaria et al. (2021)

## Preventative Strategies

As with any infectious agent, the best course of prevention is to ensure the virus doesn’t cause disease in the first place. This may be achieved through vaccination, whereby you prevent the virus gaining access and entry to target cells or through behavioral modifications that reduce the likelihood of an individual being exposed to the virus.

### Seasonal Vaccination

The most common method of preventing infection with a viral pathogen is vaccination, and for influenza viruses this is required on a seasonal basis. At present, there are two approved platforms for influenza vaccines–1) live, attenuated influenza vaccines (LAIV), and 2) inactivated influenza vaccines (Fiore et al., 2010). While both vaccines illicit a robust hemagglutinin-directed antibody response, LAIV also has the capacity to activate T cell immunity (Hoft et al., 2011; Estrada and Schultz-Cherry, 2019). Despite broad use, vaccine efficacy is variable and susceptible to changes is circulating strains. As the vaccines predominately target the hypervariable viral hemagglutinin head, they are vulnerable to mutations (genetic drift) and as such need to be reformulated on an annual basis (Demicheli et al., 2018; Sautto et al., 2018; Estrada and Schultz-Cherry, 2019). Furthermore, the time required to manufacture vaccines may impact responses during an evolving influenza virus pandemic, highlighting the need for development of a more broadly protective universal influenza vaccine (Ellebedy and Webby, 2009; Krammer and Palese, 2013; Sparrow et al., 2021).

### Universal Influenza Virus Vaccines

In an effort to circumvent issues associated with current seasonal influenza vaccines, “universal vaccine” platforms have been under development for many years. Current vaccines for influenza viruses target the highly variable head of the viral hemagglutinin (HA) protein (Ellebedy and Webby, 2009; Xu et al., 2010; Sautto, Kirchenbaum et al., 2018; Estrada and Schultz-Cherry, 2019). By targeting highly conserved regions, we may reduce the need for seasonal vaccines (Ellebedy et al., 2014; Jang and Seong, 2019). Universal vaccines that have focused on the conserved HA stalk region have been developed in recent years for influenza A viruses (Krammer and Palese, 2013). As such, stalk-based targets appear to be a promising candidate in the hunt for a universal vaccine (Mallajosyula et al., 2015). Another highly conserved potential target is the ectodomain matrix protein 2 (M2e) found on influenza A viruses. Unlike other targets, M2e does not generally stimulate the expression of neutralizing antibodies, but instead induces non-neutralizing antibodies that trigger antibody-dependent cell-mediated cytotoxicity (Simhadri et al., 2015). There has been significant activity in the universal vaccine space and candidates are under development or in clinical trial, with only limited knowledge as to the degree and longevity of protection they offer (Corder et al., 2020).

### Public Health Measures

During the current coronavirus disease (COVID-19) pandemic, governments around the world have introduced necessary public health measures in an effort to reduce the rate of transmission of SARS-CoV-2 virus. While the specific nature of these measures varied, curfews, lockdowns, social distancing and the implementation of mask-wearing have all been introduced to mitigate spread of disease. As a consequence of these measures, there has been a significant decline in the number of laboratory-confirmed influenza virus infections observed (Yeoh et al., 2020). The most likely explanation for this marked reduction is not fewer diagnostic tests, but rather the similarities in transmission route for both respiratory viruses (Wu et al., 2020; Yeoh et al., 2020). Therefore, by implementing public health measures to stem the rate of coronavirus infection, a positive byproduct has been a reduction in transmission of influenza viruses. Despite the success of these measures, it should be noted that curfews and lockdowns are simply not sustainable beyond a pandemic setting, due to the significant economic and psychosocial burden they create (Dubey et al., 2020).

## Pharmacological Intervention

Though the best course of action is to prevent influenza virus infection, as with many infectious agents, this is not always feasible. In these cases, it is necessary to switch from a preventative model to one of harm minimization. There are a range of pharmacological interventions that may be implemented as supportive therapies in order to reduce the rate of viral replication, as well as to ameliorate the damaging effects of immunopathology on tissue during the process of viral clearance. The most common of these treatments are discussed further throughout this section.

### Antiviral Drug Therapies

Following infection with influenza viruses there are several antiviral drugs that may be administered as supportive therapies. Of these drugs, there are currently three major classes approved for use in a clinical setting–M2 ion channel inhibitors, neuraminidase inhibitors, and transcriptional inhibitors (Amarelle et al., 2017; Hayden et al., 2018). It should also be noted that there are a number of drugs targeting various components of influenza viruses or the human immune system, that are currently under development and are not discussed as they are beyond the scope of this review.

#### M2 Ion Channel Inhibitors

The oldest group of antiviral drugs approved for the treatment of influenza A virus infections are the M2 ion channel inhibitors. The drugs amantadine and rimantadine inhibit the function of the M2 ion channel of influenza A viruses, preventing viral uncoating and the transfer of viral genomic material into the host cell (Hay et al., 1985; Lampejo, 2020). Due to their mechanism of action, these drugs are only effective against influenza A viruses, as all other influenza viruses lack M2 ion channels. The M2 ion channel inhibitors are no longer recommended for administartion due to the large percentage of circulating influenza A strains where resistance has been identified (Hussain et al., 2017).

#### Neuraminidase Inhibitors

Neuraminidase inhibitors are among the most effective antiviral drugs used in the clinic for the treatment of influenza virus infection. Mechanistically, these antiviral therapeutics block the enzymatic activity of the viral neuraminidase, preventing cleavage of sialic acid residues and release of new virions from the host cell (Sidwell et al., 1998). Within this class, of drugs there a currently two approved antivirals administered for the prevention and treatment of influenza A and B infection; oseltamivir (oral), and zanamivir (inhaled), and two approved for treatment only; peramivir (intravenous), and laninamivir (inhaled) (Amarelle et al., 2017; Higashiguchi et al., 2018; Gaitonde et al., 2019). Despite their success in the past, emergence of viral resistance to these drugs is becoming an increasing problem (though not to the same extent as the M2 ion channel inhibitors), and these drugs will need to be used responsibly to ensure their effectiveness is maintained (Lackenby et al., 2011; Li et al., 2015).

#### Transcriptional Inhibitors

Transcriptional inhibitors are a broad category of antiviral drugs that inhibit viral replication pathways, by blocking viral transcription. This category includes the viral RNA-dependent RNA polymerase inhibitor, favipiravir, as well as the more recent selective cap-dependent endonuclease inhibitor baloxavir marboxil (Furuta et al., 2013; Takashita et al., 2018). While both these drugs are approved for the treatment of influenza A and B infections (pandemic use only in the case of favipiravir), there is little evidence in the literature that supports their potential as prophylactics (Lampejo, 2020). Furthermore, as with other antivirals on the market, there is strong evidence to suggest influenza viruses have the potential to develop resistance to these treatments, rendering them ineffective (Goldhill et al., 2018; Takashita et al., 2019).

### Corticosteroid Treatment

Of the pharmacological interventions currently available for treatment of acute respiratory infections such as influenza viruses and SARS-CoV-2, corticosteroid therapy is possibly one of the most controversial. During infection, the immune system may respond excessively in an attempt to clear the infectious agents. In doing so, tissue may become damaged as a consequence of excessive production of proinflammatory cytokines Li et al. (2017), leading to further complications. The principle aim of this type of therapy is to counteract excessive inflammation and reduce immunopathology (La Gruta et al., 2007). Although corticosteroids have been used in the treatment of severe viral respiratory infections and associated complications for some time, evidence is beginning to emerge that administration of corticosteroids such as dexamethasone during influenza virus infection does not reduce the risk of further complications, and may in fact be contributing to increased mortality (Viasus et al., 2011; Ni et al., 2019; Ye et al., 2020; Zhou et al., 2020).

## Clinical Interventions

Despite the availability of pharmacological therapeutics for the treatment of influenza virus infection, in severe cases or in individuals with secondary complications, more invasive clinical interventions may be necessary to maintain respiratory function. The most common of these supportive measures are noninvasive mechanical ventilation, invasive mechanical ventilation, and extracorporeal membrane oxygenation (ECMO). Noninvasive mechanical ventilation refers to the provision of respiratory support without direct tracheal involvement, in the form of oxygen delivery *via* face mask, helmets, mouth pieces etc. (Popat and Jones, 2012). Conversely, invasive mechanical ventilation involves direct access to the trachea, typically via intubation (Popat and Jones, 2012). Although both measures may be necessary to preserve life, they are associated with increased risk of transmission of respiratory viruses to healthcare workers, due to the production of aerosols (Esquinas et al., 2014; Arulkumaran et al., 2020; Ng et al., 2020; Al Lawatis et al., 2021). If hypoxia continues in the presence of invasive mechanical ventilation, extracorporeal membrane oxygenation (ECMO) may be implemented as a last resort. ECMO acts as an artificial heart and lung, externally oxygenating the blood, then returning it to the circulatory system. Given the extremely invasive nature of the procedure and the many associated risks, ECMO is only ever used in life-threatening infections when all other measures have failed (Davies et al., 2009; Kowalewski et al., 2020).

## Therapeutic Potential of the Extracellular Matrix

As highlighted throughout this review, current preventative strategies and therapeutic treatments are all either not sustainable, highly invasive, or are susceptible to being rendered ineffective through constant mutations in the genome of these viruses. These factors clearly necessitate the development of novel therapeutics that counteract the vulnerabilities described throughout this review. Although the host immune response has been studied in depth as a potential source of therapeutic targets, “non-traditional” elements of the response are often overlooked.

### Extracellular Matrix

One such element often overlooked is the extracellular matrix, or ECM. The ECM is a highly dynamic, non-cellular structure that plays a key role in cellular signaling, growth, repair, migration, and general homeostasis (Hynes, 2009; Bonnans et al., 2014; Theocharis et al., 2019). In mammalian systems, the ECM is comprised of hundreds of different proteins including glycoproteins, collagens, and proteoglycans, just to name a few (Hynes and Naba, 2012). While historically viewed as a rigid network with a purely structural role, the ECM is now understood to be a highly complex and vital component of tissues. More recently, the ECM has been found to have both positive and negative associations with different disease states, depending on the degree of remodeling by metalloproteinases (Bradley et al., 2012; Klose et al., 2013; Cal and Lopez-Otin 2015; Binder et al., 2017; Herrera et al., 2018). It is therefore a logical progression to investigate the ECM and the enzymes that remodel it as a potential therapeutic target to enhance the immune response against infectious disease, including influenza virus infections.

### A Disintegrin-like and Metalloproteinase with Thrombospondin Motifs

Of these ECM remodeling enzymes, the a disintegrin-like and metalloproteinase with thrombospondin motifs (ADAMTS) family is one of the more recently discovered, with its first member ADAMTS1, identified in 1997 (Kuno et al., 1997; Porter et al., 2005). Since that time, an additional 18 mammalian members have been reported (Apte, 2009; Dubail and Apte, 2015). The ADAMTS family are responsible for cleaving a variety of substrates from proteoglycans (proteoglycanase clade), to clotting enzymes (ADAMTS13), to yet unidentified substrates (orphan clade) (Kelwick et al., 2015). As our knowledge of these enzymes has become more sophisticated, their importance in disease states has become more apparent. This has been eloquently presented in reviews by (Mead and Apte, 2018; Santamaria, 2020). While the role of these enzymes in cancer biology has been known for some time Kumar et al. (2012a), Kumar et al. (2012b), Cal and Lopez-Otin (2015), Chen et al. (2018), very little was known about their function in the immune response to viral infection. In 2016, a study by McMahon et al. (2016) found that expression of ADAMTS5 was necessary for optimal CD8^+^ T cell immunity at effector sites in the periphery, as mice lacking this enzyme displayed increased disease severity and poor viral clearance associated with an accumulation of the ADAMTS 5 substrate versican in draining lymph nodes following influenza virus infection. In contrast, a recent study by Boyd et al. (2020) demonstrated that expression of ADAMTS4 in lung fibroblasts was associated with immunopathology and poor disease outcomes in mice and humans (Boyd et al., 2020). Furthermore, systems biology approaches have indicated that overexpression of versican, an ADAMTS enzyme substrate, may be associated with hyperinflammatory responses following severe COVID-19 infection (Jung et al., 2021). These findings suggest that ADAMTS enzymes and related pathways may be attractive therapeutic targets and further investigation is warranted. In support of these findings a number of antibodies and small molecule inhibitors (for example GSK2394000, GSK2394002, 237-53, sugar-based arylsulfonamide 4b, and Agg-523) are currently available and in clinical and preclinical trials that can be used to target ADAMTS enzymes (Dancevic and McCulloch, 2014; Larkin et al., 2015; Santamaria et al., 2015; Shiraishi et al., 2016; Balchen et al., 2018; van der Aar et al., 2018; Malemud, 2019; Santamaria and de Groot, 2019; Santamaria, 2020; Santamaria et al., 2021). It should be noted however that over-expression of ADAMTS enzymes may also improve disease outcomes (McMahon et al., 2016).

### Matrix Metalloproteinases

In addition to the ADAMTS family, another key remodeling enzyme family in the ECM are the matrix metalloproteinases (MMPs). Unlike the ADAMTS family, the MMPs have been studied for quite some time with proteolytic activity first being described in 1962 (Gross and Lapiere, 1962). Of the 28 reported MMPs, 23 are expressed in humans Cui et al. (2017), Raeeszadeh-Sarmazdeh et al. (2020), and are grouped into broad clades based on structure, function and substrate preference (Raeeszadeh-Sarmazdeh et al., 2020). Unlike ADAMTS enzymes, the role of MMPs is well-documented in immune responses, and has been specifically characterized following influenza virus infection. While some family members have been associated with protection, other family members have been associated with poor outcomes following influenza virus infection. A recent study by Rojas-Quintero et al (2018) found that an absence of MMP9 in mice resulted in increased viral clearance from the lung, likely due to the induction of more effective adaptive immune responses and reduced lung damage associated with reduced E-cadherin shedding (Rojas-Quintero et al., 2018). Conversely, by inhibiting the wnt pathway and reducing the expression of MMP2 and MMP9, the extravasation of T cells is greatly impaired (Wu et al., 2007). While this may be beneficial in certain disease states where an anti-inflammatory environment is desired, in the context of influenza virus infection, reduced T cell migration can lead to delayed viral clearance and poor disease outcomes (McMahon et al., 2016). MMP inhibitors have also been tested in phase I clinical trials and have the potential to be utlized to counteract poor virus infection outcomes (Brown 1999; Fingleton 2008; Li, Saji et al., 2013).

## Conclusion

As demonstrated throughout this review, there are significant gaps and controversies surrounding current influenza virus prophylactics and therapeutics. A common theme highlighted herein is the vulnerability of our current strategies due to our overreliance of targeting influenza virus structures and replication. If we are to overcome these vulnerabilities, the development of novel therapeutics that target the host and work in consert or complement existing strategies is critical in order to prepare us for future epidemics or pandemics. The untapped potential of the ECM and its remodeling enzymes has been demonstrated herein, and further investigation of possible applications to viral immunity is warranted.
